# Preliminary Support for a Technology-Enhanced Suicide Prevention Intervention for College Students: Mixed Methods Preimplementation Study

**DOI:** 10.2196/79537

**Published:** 2026-09-09

**Authors:** Jocelyn I Meza, Joan R Asarnow, Natalia Jaramillo, Juliane L Martinez, Candice Biernesser, Brandie George-Milford, David Brent

**Affiliations:** 1 Department of Psychiatry and Biobehavioral Sciences, University of California, Los Angeles, 300 Medical Plaza Driveway Room 1235, Los Angeles, CA, 90096, United States, 1 310-825-6652; 2 Department of Psychiatry, University of Pittsburgh, Pittsburgh, PA, United States

**Keywords:** suicide prevention, young adults, mobile intervention, college students, safety planning, caring contacts

## Abstract

**Background:**

Self-injurious thoughts and behaviors, including suicidal ideation, planning, attempts, and nonsuicidal self-injury, are a significant public health concern among college students. Community college students face elevated mental health risks and persistent barriers to traditional services, particularly those from underserved and ethnoracially minoritized backgrounds, underscoring the need for scalable, accessible digital mental health interventions.

**Objective:**

This study conducted a mixed methods evaluation of the initial reactions of college students to a novel technology-enhanced suicide prevention intervention (TE-SPI), with particular focus on feedback regarding a mobile app prototype (BRITE) designed to support coping skill use and mood monitoring, as well as proposed nondemanding caring contact text messages.

**Methods:**

A mixed methods approach using the technology acceptance model (TAM) was used to assess the TE-SPI among 20 ethnoracially diverse college students (mean age 22.8, SD 3.9) from a community college (n=10) and a 4-year university (n=10). Participants were shown the BRITE app prototype on a study iPad and asked to provide feedback on its perceived ease of use, usefulness, and relevance to student needs. Quantitative measures assessed initial impressions of satisfaction and usability, while qualitative analyses examined participants’ reactions to the app prototype and preferences regarding potential caring contact text messages.

**Results:**

Participants generally reported favorable initial impressions of the BRITE app prototype, with 90% (18/20) describing it as helpful and 85% (17/20) user-friendly; 90% (18/20) also indicated that they would recommend it to peers in distress. Qualitative feedback supported these findings, highlighting the app’s perceived accessibility, relevance to student life, and potential usefulness during moments of distress. Participants also offered concrete suggestions for app refinements (eg, mood scale customization, gamification, and language options) and for useful caring contact messages they would want to receive. In total, 55% (11/20) of participants said that they would want to receive text messages as part of TE-SPI, while 25% (5/20) reported maybe or possibly wanting to receive messages.

**Conclusions:**

Findings offer preliminary insight into how college students perceive the BRITE app prototype and proposed caring contact message, highlighting concrete feedback for app refinement prior to real-world testing. This study provides early user feedback that can inform further development of the TE-SPI model. Future research should examine actual use, acceptability, feasibility, and clinical impact among diverse college students.

## Introduction

### Background

Suicide is the second leading cause of death among college-age adults [1]. Additionally, self-injurious thoughts and behaviors (SITBs)—encompassing both thoughts and behaviors involving suicidal intent, as well as deliberate self-injury without intent to die—constitute major public health issues. Data show that in the United States, 11% of college students have experienced suicidal ideation, 5% have made a suicide plan, and 2% have attempted suicide in the previous 12 months [2], and self-harm ranked third for young people ages 10‐24 years in the most recent Global Burden of Disease Survey [3]. Each year approximately 93,000 young adults (ages 18‐24 years) present to emergency departments for SITBs [4]. Furthermore, suicide rates for this age group have remained particularly high over the last decade and have disproportionately increased among ethnoracially minoritized groups in the United States [1,5]. Previous research identifies elevated symptoms of anxiety and depression as key risk factors associated with the risk of suicide among college students [6,7], particularly if left untreated [8].

### Mental Health Challenges in College Students

College students represent a diverse group of students with varying mental health profiles and needs depending on individual characteristics, socioecological characteristics, and college contexts underscoring the importance of considering student context and cultural relevance when developing mental health interventions for this population. Among the college student population, the subgroup of students attending community colleges reports higher traumatic life events, poorer physical health, and more severe psychological concerns compared to students at 4-year institutions [9-11]. Ethnoracially minoritized students and low-income populations, who make up a significant portion of the community college student body, are also less likely to engage with behavioral health services and are more likely to drop out of treatment [12-15]. Community college students who transfer to 4-year academic institutions also experience elevated risk for mental health outcomes, including SITBs, because they face several stressors as they navigate new campus climates, build social networks, and adjust to academic expectations at 4-year academic institutions [16,17]. In fact, data show that transfer students report significantly higher levels of depression, social anxiety, and educational and family-related distress compared to nontransfer students [18]. Contextualizing treatments to address the needs of community college students, including those who transfer to 4-year institutions, can lead to higher treatment engagement.

The diverse mental health challenges faced by community college students, along with limited access to resources, highlight the urgent need for innovative suicide prevention strategies. A few of the common barriers faced by community college campuses include financial restrictions, staff shortages, long waiting lists, vague and unstandardized crisis response guidelines, and limited student outreach [9,19,20]. In addition, some students choose not to report their mental health symptoms or seek professional support during a crisis as a result of perceived stigma, apprehension, or feeling that they may be a burden. To address this gap, novel evidence-based digital mental health interventions present a promising solution, particularly for underresourced community colleges.

### Digital Mobile Apps for Suicide Prevention

Digital mental health interventions may bolster mental health care on college campuses and can promote skills that mitigate risk for SITBs [21-23]. Mental health apps have potential for offering timely support, especially given widespread reports of time constraints, perceived inconvenience associated with traditional face-to-face services, and the high levels of comfort and acceptance of technology among students [15,21,22,24]. Digital tools also have the potential to reduce suicide risk by providing discreet support for individuals struggling with mental health issues who may be hesitant to seek out resources and can offer a range of intervention components, including support for crisis intervention, mood tracking, and coping strategies, which can help users manage their symptoms [25]. Additionally, digital tools allow for continuous monitoring and support, offering timely interventions during moments of crisis. By integrating features such as direct links to crisis hotlines and emergency contacts, digital tools can ensure that users receive the necessary assistance promptly. From a research standpoint, user data collected can also offer objective insights on usability, guiding iterative updates and improvements. Overall, digital mental health tools can play a crucial role in suicide prevention by making resources more accessible and tailored to individual needs.

Despite the rapid growth of digital mental health tools, few are designed with the unique needs of college students in mind. In particular, rigorously tested apps for suicide prevention have primarily focused on adolescents who present to acute care settings. For example, BlueIce, an app with mood ratings and personalized features to improve mood (ie, using cognitive behavioral therapy and dialectical behavior therapy skills), was specifically codeveloped with adolescents with lived experience, and the phase 1 open uncontrolled trial found that nearly three-quarters of participants had reductions in self-harm at 12-week follow-up [26]. Similarly, not all evaluations of suicide prevention apps include ethnoracially minoritized samples, with the exception of the iBobbly app, a suicide prevention app for Indigenous Australian youths and young adults [27]. As Ramos et al [15] highlight, most existing app evaluation frameworks fail to account for critical diversity, equity, and inclusion factors such as literacy, cultural relevance, and technology access, which limits the effectiveness and equity of digital interventions. This gap underscores the need for more inclusive, accessible, and clinically guided digital tools. Indeed, community college students remain significantly underrepresented in suicide research, particularly around research that focuses on the development of digital mental health and suicide prevention tools, underscoring a significant gap and the urgency to address current SITB disparities for at-risk college students [28].

### Technology-Enhanced Approach to Suicide Prevention

Combining the promise of digital interventions with a “human touch” has shown promise for increasing use of digital interventions, an important goal given that low use limits intervention exposure and benefits and has been a problem in trials of prior interventions [29-32]. In an effort to optimize intervention use and value, we pilot-tested a technology-enhanced suicide prevention intervention (TE-SPI). Through TE-SPI, we aimed to adapt a previously validated safety planning and emotion regulation smartphone app, initially designed for adolescents [25,33,34], for use among college students. To increase engagement within this population, we added a new component, caring contact text messages, which are nondemanding messages of care designed to increase feelings of connection and support postcrisis [35-37]. Caring contacts have demonstrated high feasibility and acceptability among adults when delivered via email or text [38,39]. Although some data indicate a 1-year benefit in reducing self-reported suicide attempts [37,40], evidence remains mixed regarding its effect on suicide mortality or self-harm–related emergency department and hospital visits. Moreover, there is limited research on its effectiveness among diverse college student populations. Therefore, this project presents formative prototype evaluation data based on participant feedback to inform the adaptation and refinement of 2 synergistic intervention components for college students at risk for SITBs.

### Technology Acceptance Model

The technology acceptance model (TAM) has been widely used to study the implementation of new technologies and digital tools, including pilot trials for mental health apps [41,42]. The TAM framework, based on the theory of reasoned action [43], posits that an individual’s behavior (ie, use of a digital tool) is influenced by their attitudes and perceptions. We used TAM as a guiding framework to understand how college students perceived the components of a TE-SPI. More specifically, according to the TAM, 2 key factors predict an individual’s engagement and use of new technology: perceived ease of use and perceived usefulness [41]. Perceived ease of use refers to how easily a user believes that they can learn and use the technology without difficulties, while perceived usefulness pertains to the belief that the new technology can enhance their daily routines [41]. However, an individual’s perception of new technology can also be significantly shaped by their social environment and context [44]. For instance, perceptions may change based on friends’ or family members’ reactions and experiences with the digital tool, and community college students might find digital tools more beneficial if they are contextualized to their experiences and needs.

### This Study

This mixed methods study aimed to explore the perceptions of college students of a TE-SPI designed to reduce distress and improve mental health well-being. We used the TAM framework to examine the perceived ease of use and perceived usefulness of the TE-SPI. The primary research questions guiding the study were:

- Question 1: What are community college students’ perceptions of the TE-SPI features?
- Question 2: What is the perceived usability of the TE-SPI? (ie, what are the aspects and factors, in terms of functionality and utility, that students find useful?)
- Question 3: What aspects of the TE-SPI do students find more engaging?
- Question 4: What factors may influence the decisions of community college students to use the TE-SPI?

## Methods

### Research Design

#### Procedures and Participants

This substudy is part of a large research study examining the Screening and Treatment for Anxiety & Depression (STAND) system of care in a community college setting. The STAND system of care was developed as part of the University of California, Los Angeles (UCLA) Depression Grand Challenge to be a scalable approach to preventing, identifying, and treating anxiety and depression [45]. The STAND study was approved by the institutional review board at UCLA (IRB #22‐0205). Participants were recruited from a local community college (East Los Angeles Community College [ELAC]) and a Transfer Student Center for community college students at a 4-year university (UCLA), enhancing student diversity over that afforded by a single community college site. Recruitment was from the fall of 2022 through the winter of 2023. Participants from ELAC were recruited through a listserv of students interested in participating in STAND-related research studies, and UCLA participants were recruited using outreach through informational presentations and flyer postings at the Transfer Student Center. Study eligibility criteria included (1) currently enrolled at ELAC or UCLA as a transfer student, (2) owning a smartphone, and (3) English proficiency. Eligible students interested in participating were screened on the phone and provided oral consent. Participants then scheduled an interview date with the study staff to complete the semistructured interview and brief surveys. All participants received a US $25 e-gift card for their participation in the study.

To ensure participant safety and confidentiality, participants were informed during the consent process that they could decline to answer any questions or stop the interview at any time without penalty due to the sensitive nature of the study topics (eg, distress, depression, and anxiety). Research staff conducting the interviews were trained to recognize signs of distress and to pause or discontinue the interview if participants became notably distressed. Research staff were also trained to assess the participant’s well-being and offer additional support and mental health resources to all participants. Additionally, limits of confidentiality were clearly communicated to participants, including mandated reporting requirements related to risk of harm to self or others. A safety protocol was activated when participants disclosed current active suicidal ideation, particularly with intent or plan. The safety protocol included a risk assessment and safety planning by a licensed clinical psychologist, although there were no cases in this study that required the use of the safety protocol.

In total, 14 students from ELAC and 13 students from UCLA were eligible to participate in the study and provided oral consent. A total of 4 participants from ELAC and 3 from UCLA were excluded from the study due to failure to attend their scheduled semistructured interviews. Therefore, 20 participants were included in the analysis. Participants (N=20) in the study were between the ages of 19 and 32 (mean 22.8, SD 3.9) years. The sample consisted of both community college students (n=10) at ELAC and transfer students at UCLA (n=10).

#### Data Collection

College students participated in the study in person or virtually using a secure videoconferencing platform (Zoom, UCLA Health System Protected). Participation consisted of completing a semistructured interview and 2 brief online surveys with a research staff. Semistructured interviews lasted up to 45 to 60 minutes each (average time was approximately 36, SD 12.8 minutes) and were audio-recorded and stored in a secure UCLA Box folder. Prior to starting the semistructured interview, participants were asked to complete the first brief online survey administered via Qualtrics inquiring about their demographic background, mental health use, and previous digital tool use for mental health concerns. Next, participants were introduced to the technology TE-SPI components, specifically (1) the BRITE app, which includes mood and distress ratings, a menu of distress tolerance and emotion regulation skills [25,33], and a coping or safety plan that students can turn to when needed to cope effectively (eg, when distress is high or increasing); and (2) caring contacts (brief nondemanding text messages aimed at providing connection to the intervention team and support [46]). As part of the prototype demonstration, participants were informed that both the BRITE app and caring contacts are designed to be introduced by a mental health provider. Next, the participants were given a study iPad and received a demonstration of how they would be onboarded to get access to the app (ie, after completing a safety plan with a clinician, although the development of a safety plan was not conducted as part of the demonstration). Then, students were given a tutorial on how they could create and individualize their account or profile to use the various features including (1) mood ratings, (2) use of coping activities, and (3) how to activate the support feature. Following this process, participants were given time to explore the app on the study iPad and were encouraged to ask questions regarding app features. After having time to review the app, participants were asked to share their perceptions and feedback on the app features. Next, the concept of caring contacts was presented with various examples, and participants were asked to share their perceptions and feedback on the BRITE app and caring contact TE-SPI components. Feedback for the clinician session to develop a safety plan was not included as part of the feedback, given its already established feasibility and acceptability in college samples [47]. Throughout the interview process, the research staff used a semistructured interview protocol to guide the participant through each of the questions. Following the interview, participants completed the second brief quantitative survey inquiring about their perceptions of the TE-SPI components and overall satisfaction questions. At the end of the study, all participants were sent information regarding campus and community mental health resources and received a US $25 e-gift card as compensation for participating in the study.

### Ethical Considerations

This substudy was conducted separately from the STAND trial and reviewed and approved by the institutional review board at UCLA (IRB #22‐0244). All procedures complied with institutional and federal ethical standards. Informed consent was obtained from all participants prior to study participation. Participants were informed that their participation was voluntary and that they could decline to participate or withdraw from the study at any time without penalty. Additionally, to protect participants’ privacy and confidentiality, all identifying information was removed from the study data prior to analysis.

### Materials and Measures

#### TE-SPI Materials

##### BRITE App

The BRITE app includes (1) the development of a coping plan with a clinician, designed to support effective coping strategies that include evidence-based suicide prevention intervention tools; (2) mood and distress ratings; (3) a menu of distress tolerance and emotion regulation skills; and (4) a reaching out feature for support during distress. On the BRITE app, users are prompted to rate their mood or distress daily, but they can also self-initiate their own ratings. After entering a mood and distress rating into the app, users are provided with potentially helpful skills and activities (ie, mindfulness and distress tolerance skills) that they can complete independently to improve their mood or reduce their level of distress. If participants choose to complete the suggested activities, they are asked again to rate their mood and distress level after completing the suggested activities. Participants can personalize their profiles in the BRITE app by uploading photographs, videos, or links to websites and can update their coping plan with individualized warning signs, coping skills, and phone contacts for interpersonal support and clinician contact. Although participants were not asked to download the BRITE app onto their phones, they were given time to review all features of the tool via an iPad that was provided to them for use during the interview. See Kennard et al [25,33] for more details on the development of the digital tool (BRITE app), which was primarily developed for adolescents in acute care settings (ie, inpatient hospitalization).

##### Caring Contacts

Additionally, the TE-SPI model adds caring contacts in addition to the BRITE app to create a unified platform. Caring contact messages are brief, low-cost, nondemanding expressions of care—typically delivered via text, email, phone, or mail (eg, “Hey there, I’m wishing you well. I hope you’re taking good care of yourself.”)—and are traditionally sent by a care provider or care team to remind individuals that support is available and to promote connection [36,46]. As part of the TE-SPI model, the concept of caring contacts was introduced to participants. In this study, the study interviewer explained the approach for these messages as well as who and when these messages would be sent. Students were also provided with example messages such as “Hey, I’ve enjoyed working with you. Hope your day is going okay so far.” Students were then asked about their perceptions of this approach, whether they would like to receive such messages, and were asked to share an example of a caring contact message that they would find meaningful and helpful during moments of distress.

### Measures

#### Mental Health Status

Current symptoms of depression and anxiety were assessed using the Patient Health Questionnaire 2-item (PHQ-2) and the Generalized Anxiety Disorder 2-item (GAD-2 [47,48]). Both the PHQ-2 and GAD-2 measures were used to descriptively characterize the mental health symptom profile of the sample and were not used for hypothesis testing or subgroup analyses. The PHQ-2 is a self-report measure of depression symptoms over the last 2 weeks. The 2 items from PHQ-2 were the following: “Over the last 2 weeks, how often have you been bothered by having little interest or pleasure in doing things?” and “Over the last 2 weeks, how often have you been bothered by feeling down, depressed, or hopeless?” Response options ranged from 1=not at all to 4=nearly every day. The measure has good internal consistency and concurrent validity [47,49]. The GAD-2 is a self-report measure of anxiety symptoms over the last 2 weeks. The GAD-2 includes the following 2 items: “Over the last 2 weeks, how often have you been bothered by feeling nervous, anxious or on edge?” and “Over the last 2 weeks, how often have you been bothered by not being able to stop or control worrying?” Response options ranged from 1=not at all to 4=nearly every day*.* The measure has strong psychometric properties and has been found to be reliable [47,49]. Both items from the PHQ-2 and GAD-2 were summed separately, and a score of 3 was used as the cutoff for identifying possible cases of depression and generalized anxiety disorder, respectively [47,48,50]. The PHQ-2 (α=.826) and GAD-2 (α=.928) measures have strong internal consistency in our sample.

#### Feasibility and Acceptability

Feasibility and acceptability were assessed using both quantitative and qualitative indicators, including participant ratings of usability, satisfaction, perceived usefulness, engagement with app features, and qualitative feedback regarding BRITE app and caring contacts’ relevance, accessibility, and suggested refinement.

#### Technology Use

To examine participants’ overall technology use, we formulated questions to inquire about their access to a cell phone or smartphone, data plan, how often participants carry their phone, and specifics on how much time they spend on their mobile device (time measured in total minutes per day) for the following activities: texting, talking, emailing, surfing the web, playing games, social media apps, and mental health apps. Sample questions included “Do you have a smartphone?” and “How often do you carry your phone with you?.” Additionally, participants were instructed to list the mobile apps that they frequently use and the type of features they found useful.

#### Digital Intervention Barriers

The Digital Intervention Barriers Scale-7 [44] was used to assess participants’ perception of the TE-SPI, specifically their perceptions of engagement, motivations for use, and if the digital tool was adequate (not too long or too short). This measure asked students to “Please indicate the extent to which you agree or disagree with the following statements.” Statements included (1) “I didn’t understand the tasks or things I was supposed to do on the app.”, (2) “I thought the app wasn’t engaging.”, (3) “It would be difficult to keep myself motivated to use the app.”, and (4) “I thought the length of the app wasn’t adequate (too long or too short).” The answer choices participants were able to choose from for all 4 questions were 1=totally disagree, 2=disagree, 3=neutral, 4=agree, and 5=totally agree. Participant responses were scored by obtaining a sum, with higher scores indicating more barriers to future use and engagement with the digital tool. Three items from the original Digital Interventions Barriers Scale-7 were omitted, given that students were not asked to download the app during this phase of the study. The omitted items were (1) “I had technical problems with my technology (eg, device, internet, platform).”, (2) “I forgot to use the app.”, (3) “The app didn’t seem to be helping me.” This measure has been found to have high internal consistency and criterion-related validity [44]. The internal consistency of the 4 items was high (α=.783).

#### Satisfaction With the BRITE App Component of TE-SPI

Using a questionnaire developed by the University of Pittsburgh [51], we assessed participants’ satisfaction of the digital tool, by administering the following questions: (1) “If you started to feel really stressed and upset, do you think you would use the BRITE app?” with the following answer choice for participants to choose from 1=no, 2=possibly, 3=probably, 4=definitely, and 5=prefer not to answer; (2) “If a friend was in need of mental health care for depression or suicidal thoughts or behaviors, would you recommend the BRITE app to help them cope?” with the following answer choices: 1=definitely not, 2=no, I don’t think so, 3=yes, I think so, 4=yes, definitely, and 5=prefer not to answer; (3) “How often do you think you would use the BRITE app if it were available to you during the month after it was introduced to you?” with the answer choices being: 1=never, 2=once or twice*,* 3=3 or 4 times, 4=5 or 10 times, and 5=more than 10 times; (4) “Do you think you would be more likely to use the BRITE app if you received messages reminding you of the app?” with the following answer choices: 1=no, I would find these messages annoying, 2=no, but the messages would not bother me, 3=maybe, 4=possibly, and 5=yes. After asking each of these questions, participants could provide additional written feedback or reasoning for their answer choices. The satisfaction with BRITE score was obtained by creating a composite score that summed all 4 questions in the measure.

#### Poststudy Satisfaction and Usability

To obtain more information regarding participants’ experience with the TE-SPI components, they answered items from the Poststudy System Usability Questionnaire [42]. The Poststudy System Usability Questionnaire has been used in previous suicide prevention trials for adolescents after a hospitalization [34]. The questionnaire consists of 6 items that identify system usability. Participants rated questions on a 7-point Likert scale, ranging from 1=strongly disagree to 7=strongly agree. The following questions were asked: (1) “Overall, I am satisfied with how easy it is to use BRITE app.”; (2) “The information provided in the BRITE app such as on-screen messages, and other documentation like links or pictures for the topic activities, was clear.”; (3) “The information provided in the BRITE app, such as notifications to rate my distress or mood with emojis, was clear.”; (4) “I like interacting with the BRITE app program.”; (5) “I needed to learn a lot of things before I could get going with the BRITE app.”; and (6) “The information provided, such as the coping plan for BRITE users, was clear.” This measure is reliable with a Cronbach α that spans from 0.83 to 0.96 [51,52]. A composite sum score was created, such that a higher score indicated higher satisfaction and perceived usability. An additional question was added to assess participants’ perceptions of caring contacts. Specifically, participants were asked “Do you think you would be more likely to use the BRITE app if you received text messages from your mental health provider?,” with the following answer options: 1=no, I would find them annoying, 2=no, but the messages would not bother me, 3=maybe, 4=possibly, and 5=yes.

### Data Analysis

#### Quantitative Analysis

Descriptive analyses were conducted using SPSS (version 25; IBM Corp) software. We carried out descriptive statistics to obtain frequencies and percentages, means, and medians along with SDs and ranges for all quantitative measures.

#### Qualitative Analysis

For the qualitative analyses, all interview audio recordings were auto-transcribed using Microsoft Word software, and the transcripts were then reviewed individually by 2 independent researchers (NJ and JLM) to verify accuracy. Errors from the transcripts were fixed, and any identifiable participant information was removed. Additionally, the supervising researcher (NJ) checked each transcript for errors. The transcripts were then used for data coding.

A rapid qualitative coding approach was used from the health services literature [53], which prioritizes the efficient delivery of information to guide decision-making, particularly during preimplementation phases. First, the team developed a codebook with domains that were identified by the TAM framework. Then, using deductive and inductive strategies, coders identified additional codes and themes beyond the predefined TAM domains. After the codebook was finalized, 2 trained members independently applied domain analysis and created a summary template for each transcript. After all transcripts were coded and summarized, a matrix method was used to efficiently summarize and explore themes across all interview transcripts [54]. Themes were derived through comparison across structured summaries and matrix-based synthesis. This approach generates contextually rich information while being time efficient, and it facilitates continuous improvement and adaptation of intervention development [53].

The qualitative team did not use qualitative coding software, as the rapid matrix method was conducted using structured summary templates and team-based synthesis. The team conducted weekly meetings to review interview content, compare coding decisions and interpretations, resolve discrepancies through discussion, and refine the analytic approach. Using a rapid qualitative analysis process, each interviewer independently completed a standardized summary template following each interview (Multimedia Appendices 1 and 2), which included structured domains, initial content summaries, preliminary codes, and interpretive reflections [49]. These completed templates were compiled and reviewed by 2 researchers (NJ and JLM), who collaboratively synthesized responses across participants using a matrix method approach to identify key patterns and emerging themes. These themes were iteratively refined through weekly team discussions to compare summaries and interpretations and solve discrepancies to ensure consistency and representativeness across participants. Disagreements in coding were resolved through consensus-based discussion among the qualitative research team. A second review of the interviews for supporting quotes and thematic consistency was also carried out by the research team. Themes were finalized through iterative team discussions and comparison across structured matrix summaries to ensure consistency and alignment with participant responses. Throughout this process, analytic memos documenting observations and methodological reflections were completed after each interview and integrated into final thematic interpretation to enhance rigor and contextual grounding. Given the exploratory aims of the study, the sample size was considered sufficient to identify preliminary patterns in participant perceptions and inform future refinement of the TE-SPI. The data findings were considered sufficient to identify preliminary patterns relevant to intervention refinement. Furthermore, the analysis prioritized identification of salient patterns in participant feedback rather than quantification of theme prevalence.

The identified category themes were organized according to the TAM [41]. These themes helped focus the analysis, ensuring that key aspects of the data were systematically examined and aligned with the research questions. The research team defined and named the themes based on TAM (ie, perceived ease of use and perceived usefulness), then connected relevant quotes to these codes and organized them according to the model. The final step of the data analysis process was to produce a concise summary to address the research questions.

## Results

### Descriptive Analyses

Participants (N=20) in the study were between the ages 19 and 32 (mean 22.8, SD 3.9) years. The sample consisted predominantly of female participants (14/20, 70%). Participants were community college students (n=10) at ELAC and transfer students at UCLA (n=10). In terms of academic standing, 5% (1/20) of the participants were freshmen, 25% (5/20) were sophomores, 50% (10/20) were juniors, 5% (1/20) were seniors, and 1 participant preferred not to answer. For ethnicity, 55% (11/20) of participants identified as Hispanic or Latinx, and 45% (9/20) identified as non-Hispanic or Latinx; for race categories, 10% (2/20) identified as American Indian or Alaska Native, 25% (5/20) as Asian, 25% (5/20) as White, 20% (4/20) as multiracial, 10% (2/10) as other, and 2 students preferred not to answer the race category question. Data on participants’ ethnicity and racial background were assessed separately. Participants who identified as Hispanic or Latinx may also be represented within the racial categories. Background information was also collected, including previous treatment use and previous mental health app use. Half of the sample (10/20, 50%) reported that they had obtained counseling and therapy or seen a psychiatrist for their mental health in the past 12 months. Furthermore, participants (3/20, 15%) reported that they had used a digital tool for their mental health in the past. See Table 1 for additional demographic information.

**Table 1.** Participant demographics and characteristics (N=20)^a^.

Variables	Values, n (%)
Age (years)	
19‐25	16 (80)
26‐32	4 (20)
Gender identity	
Male	5 (25)
Female	14 (70)
Nonbinary or third gender	1 (5)
Ethnicity	
Hispanic or Latine/x	11 (55)
Non-Hispanic or Latine/x	9 (45)
Race	
American Indian or Alaska Native	2 (10)
Asian	5 (25)
White	5 (25)
Other	2 (10)
Multiracial	4 (20)
Prefer not to answer	2 (10)
College standing	
Freshman	1 (5)
Sophomore	5 (25)
Junior	10 (50)
Senior	1 (5)
Prefer not to answer	3 (15)
University or community college student	
Transfer student	10 (50)
Community college	10 (50)
US born	
No	2 (10)
Yes	18 (90)
Mental health scores	
PHQ-2^b^ (score of 3 and above)	4 (20)
GAD-2^c^ (score of 3 and above)	8 (40)
Received mental health treatment	
Yes	10 (50)

a Ethnicity was assessed separately from race. Participants who identified as Hispanic/Latinx may also be represented within the racial categories.

b PHQ-2: Patient Health Questionnaire 2-Item.

c GAD-2: Generalized Anxiety Disorder 2-Item.

Regarding the mental health status of the participants in this study, data on self-reported symptoms indicated a mean composite score of 1.75 (SD 2.00) on the PHQ-2 (depression) and 2.45 (SD 1.82) on the GAD-2 (anxiety). A total of 4 (20%) participants were above the PHQ-2 screening threshold, suggesting possible elevated symptoms of depression and/or anhedonia, and 8 (40%) students scored above the GAD-2 threshold, indicating possible elevated anxiety symptoms. In total, 12 (60%) students scored above the screening threshold, indicating possible elevated symptoms for anxiety or depression. These results are presented to provide context regarding the sample’s mental health profile and were not used to examine the differences in the perceptions of the intervention.

### Perceived Ease of Use: BRITE App

#### Overview

This study examined initial user perceptions of the TE-SPI components using the TAM framework. Most participants perceived the BRITE app prototype as easy to interact with, with 75% (15/20) strongly agreeing and 5% (1/20) agreeing that they understood how to engage with it. Additionally, 85% (17/20) found the app features engaging (totally agreed: 13/20, 65% and agreed: 4/20, 20%). Potential motivation to use the app varied, with 50% (10/20) strongly agreeing, and 20% (4/20) agreeing that they would stay motivated to use it if they downloaded the app on their phone, while 15% (3/20) remained neutral, and 15% (3/20) disagreed. Regarding the app’s feature set, 65% (13/20) strongly agreed, and 15% (3/20) agreed that it provided an adequate number of resources. See Table 2 for additional information on participant ratings.

Participants also rated their initial satisfaction with the app’s ease of use: 35% (7/20) strongly agreed, 40% (8/20) somewhat agreed, and 20% (4/20) agreed that the app features were easy to use, while only 5% (1/20) remained neutral. Interaction with the app was well received, with 35% (7/20) strongly liking it, 40% (8/20) somewhat liking it, and 5% (1/20) disliking it. Regarding clarity, 60% (12/20) strongly agreed, and 30% (6/20) somewhat agreed that on-screen messages and activity links were clear. For distress and mood rating notifications, 45% (9/20) strongly agreed, 40% (8/20) somewhat agreed, and 10% (2/20) remained neutral about their clarity. Similarly, 70% (14/20) strongly agreed, and 20% (4/20) somewhat agreed that the personal coping plan information was clear, while 5% (1/20) found it unclear. See Table 3 to see more student feedback that was received on the app’s perceived ease of use.

**Table 2.** Digital Intervention Barriers Scale (N=20)^a^.

Question	Please indicate the extent to which you agree or disagree with the statements				
	Totally disagree, n (%)	Disagree, n (%)	Neutral, n (%)	Agree, n (%)	Totally agree, n (%)
I didn’t understand the tasks or things I was supposed to do on the app.	15 (75)	1 (5)	—^b^	2 (10)	2 (10)
I thought the app wasn’t engaging.	13 (65)	4 (20)	1 (5)	1 (5)	1 (5)
It would be difficult to keep myself motivated to use the app.	10 (50)	4 (20)	3 (15)	2 (10)	1 (5)
I thought the length of the app (ie, number of features) wasn’t adequate (too long or too short).	13 (65)	3 (15)	3 (15)	—	1 (5)

a Three items from the original Digital Interventions Barriers Scale-7 were omitted, given that students were not asked to download the app during this phase of the study. The omitted items were (1) I had technical problems with my technology (eg, device, internet, and platform), (2) I forgot to use the app, and (3) The app didn’t seem to be helping me.

b Not endorsed.

**Table 3.** Poststudy System Usability (N=20).

Question	Please rate the statement about your experiences with the app on a scale of 1 to 7 with 1 being strongly disagree and 7 being strongly agree.						
	Strongly disagree, n (%)	Somewhat disagree, n (%)	Disagree, n (%)	Neutral, n (%)	Agree, n (%)	Somewhat agree, n (%)	Totally agree, n (%)
Overall, I am satisfied with how easy it is to use the BRITE app.	—^a^	—	—	1 (5)	4 (20)	8 (40)	7 (35)
The information provided in the BRITE app, such as on-screen messages, and other documentation like links or pictures for the coping activities, was clear.	—	1 (5)	—	—	1 (5)	6 (30)	12 (60)
The information in the BRITE app, such as notification to rate my distress or mood with emojis, was clear.	—	—	—	2 (10)	1 (5)	8 (40)	9 (45)
I liked interacting with the BRITE app program.	—	—	1 (5)	3 (15)	1 (5)	8 (40)	7 (35)
I needed to learn a lot of things before I could get going with the BRITE app.	8 (40)	6 (30)	—	4 (20)	—	1 (5)	1 (5)
The information provided, such as the personal coping plan for the BRITE users, was clear.	—	1 (5)	—	—	1 (5)	4 (20)	14 (70)

a Not endorsed.

#### Look and Feel

During the semistructured interviews, participants were asked to provide qualitative feedback on the TE-SPI components. They shared their opinions on the BRITE app prototype aesthetic elements, such as the font, color scheme, and navigation (see Table 4for sample quotes and Multimedia Appendix 3 for images of the app prototype) as well as feedback on caring contact messages that they would find helpful and useful. The overall consensus we obtained based on student feedback was that the BRITE app was perceived as user-friendly, though some students expressed a desire for a broader range of emotions in the mood and distress rating scale. Some participants noted difficulty in identifying and connecting with the emotions represented by the tool’s emojis. One student mentioned:

*I think about the distress rating thing ... it’s hard to quantify. If there were other ways expressing [the emoticon] ... or a number or a short sentence that you can put into categories to understand the displayed emotions.*

**Table 4.** Themes identified across domains^a^.

Technology acceptance model components and subtheme	Sample quote	Participants mentioning related subtheme, n (%)
Perceived ease of use		
Look and feel	“I think the colors and the fonts are good ... they’re really big ... And I like how it has like characters in it” [Transfer student]	16 (80)
Look and feel	“I think it was easy to navigate in general because you can just go on the menu, and then you can find exactly what you’re looking for” [Transfer student]	11 (55)
Look and feel	“I think it’s a fun app, it definitely feels like it could make an impact” [Transfer student].	2 (10)
Perceived accessibility	“If I don’t have like a like a social support system that I can rely on at that moment, and if I feel like I need to somehow reframe my thoughts or ... just ease the mind a bit from like the negative stuff that’s going on in my mind, I will use it personally” [Community college student].	1 (5)
Perceived accessibility	“I would use the [BRITE app] because it’ll be more accessible and since I already carry my phone on a daily basis, I could use it while waiting for transportation, a class gap ...” [Transfer student].	6 (30)
Perceived usefulness		
Tool use in moments of distress	“Maybe just when I’m feeling down ... I can just go back to the app ... Also the stuff like breathing, sleeping, and exercising ... maybe it will just remind me to these things and to distress from everything” [Transfer student]	3 (15)
Tool use in moments of distress	“When I want to release my stress ... my emotions ... I want to tell someone how I feel ... no one wants to just look at a [digital tool] like when they’re sad” [Community college student].	6 (30)
Tool use in moments of distress	“I think we believe that going to therapy is more than enough, but maybe this could be an additional tool to therapy ... You know, therapist aren’t available 24 hours a day ... so this would be a helpful tool in those times” [Transfer student].	1 (5)
Interactive components	“I think about the distress rating thing ... it’s hard to quantify. If there were other ways expressing the emoticons ... or a number or a short sentence that can put into categories to understand the displayed emotions” [Transfer student].	7 (35)
Interactive components	“I would definitely say ... It sounds like it would be helpful. Like I think the section where you can upload images and things that are important to you. I thought that it would be pretty useful” [Community college student, female, 25 years].	3 (15)
Interactive components	“Me personally, like I’m someone who uses my phone on dark mode, so to incorporate that it would be a big thing” [Community college student].	2 (10)

a Perspectives were included from a small number of participants (eg, 1 or 2), when they offered a unique perspective that added to the conceptualization of the overall theme.

To address this, several participants suggested changing the scale by incorporating numerical values or descriptive labels for the emoticons, which would help users more accurately select their current emotions. For example, one student shared:

*Maybe adding more emotions besides these faces ... It doesn’t look like there is a neutral face and I can’t tell if one expression is crying or sleeping.*

Participants also recommended additional features to enhance the app’s functionality. These recommendations included a calendar and journal feature for tracking mood, distress, and customization options, such as adding an avatar. The app’s lack of a dark mode, which is a visual display setting that changes the light background and dark text of an interface to a dark background with light text, was another point of concern. Participants expressed the importance of this feature for nighttime use to reduce eye strain. Their comments included:

*I guess adding a dark mode, in case it’s nighttime ... It won’t sting my eyes and there are some people that get very sensitive to light.*

Another student noted: “Absolutely, these days, maybe like a dark mode interface could help.” Overall, results on perceived ease of use highlight that this sample of students reported a high level of ease in using the BRITE app. They recommended that adding features like a dark mode and expanding the emotion rating scale would significantly enhance the tool’s perceived ease of use.

#### Perceived Accessibility

Participants were also asked broadly about any sociocultural and developmentally related considerations of the BRITE app. In general, participants expressed that the overall feel of the tool felt culturally congruent and relevant to the college student context. Participants felt that young adult users could easily connect and relate to the content in the app. College students expressed that the language was straightforward and easy to understand; however, a few participants recommended adding a feature that allows users to translate the language in the app to the language they prefer to speak so that it could be more inclusive, especially for international students. Concerning this topic, a participant noted the following:

Maybe you could in the future ... translate the different words ... so that more people can feel inclined to use it. Maybe they don’t speak English, you know ... so they want to have it in their own language ... So in the beginning, when you first get on the app maybe ask, “Please select your language.”

In addition to language, some participants discussed that the BRITE app was appropriate for college students, as it was easily accessible. A participant shared the following: “I would use the[app] because it’ll be more accessible and since I already carry my phone on a daily basis, I could use it while waiting for transportation, a class gap ...”

The BRITE app’s accessibility and convenience appeared to be particularly appealing to this student population. Participants shared examples of how the app could fit naturally into their college life routine, making it easy to use during gaps of time, such as waiting for transportation or between classes. This convenience factor highlights a significant advantage of mental health mobile apps, as they can be well integrated into daily habits without requiring students to set aside much dedicated time. It is possible that the app can increase use frequency by turning typically unproductive periods into opportunities for digital mental health content interaction. Overall, this participant feedback emphasized the potential advantages of digital tools available on phones as an avenue to promote mental health engagement among community college and transfer students.

### Perceived Usefulness

#### Overview

Participants completed a usability questionnaire that provided valuable insights into their perspectives on the perceived usefulness of the TE-SPI components. In terms of use during moments of distress, half of the participants (10/20, 50%) indicated that they would definitely use the app if they felt distressed or upset. When asked if they would recommend the BRITE app to a friend needing mental health care for depression or more severe distress (ie, having thoughts of suicide), 50% (10/20) responded, “yes, I think so,” and 40% (8/20) responded “yes, definitely.” Regarding potential use in the first month of availability, 45% (9/20) of participants stated they would use the app 3 to 4 times, 35% (7/20) would plan to use it 5 to 10 times, and 15% (3/20) would expect to use it more than 10 times. When participants were asked if they would engage more with the BRITE app if they received caring contact messages, 55% (11/20) said “yes,” 25% (5/20) said “possibly or maybe,” and 20% (4/20) stated that the messages would not increase their overall motivation to use the app. When asked to elaborate on their ratings, one student mentioned:

*We’re are always connected to our phones, and at this age it is basically an extension of ourselves. I think reminders [text messages] would be helpful because notifications are a good way to get more engagement with the app*.

Another participant said: “If I was reminded, then I would have an inclination to return [to the app] since it gave me a reminder to go on,” while another stated: “I tend to forget things, it [text messages] would help to keep on track.” During the qualitative interviews, participants were also asked to elaborate on their perceptions of caring contacts by providing suggestions of caring contact messages that they would find useful (see Table S1 in Multimedia Appendix 4 for additional suggestions provided by participants). Overall, the survey feedback from students suggests a varied perception of the TE-SPI’s perceived usefulness, with a clear indication that caring contact messages could enhance user engagement, although with some reservations regarding the frequency and content of the caring contact messages. See Table 5 to see more students’ input on the perceived usefulness of the app.

**Table 5.** Satisfaction or qualitative questions (N=20).

Questions	Values, n (%)
If you start to feel really stressed and upset, do you think you would use the BRITE app?	
No	1 (5)
Possibly	4 (20)
Probably	5 (25)
Definitely	10 (50)
Prefer not to answer	—^a^
If a friend were in need of mental health care for depression or suicidal thoughts or behaviors, would you recommend BRITE to help them cope?	
Definitely not	—
No, I do not think so	1 (5)
Yes, I think so	10 (50)
Yes, definitely	8 (40)
Prefer not to answer	1 (5)
Do you think you would be more likely to use the BRITE app if you received messages reminding you of the app?	
No, I would find these messages annoying	4 (20)
No, but the messages would not bother me	—
Maybe	2 (10)
Possibly	3 (15)
Yes	11 (55)
How often do you think you would use the BRITE app if it were available to you during the month after it was introduced to you?	
Never	1 (5)
Once or twice	—
3 or 4 times	9 (45)
5 or 10 times	7 (35)
More than 10 times	3 (15)

a Not endorsed.

#### Tool Use in Moments of Distress

Participants offered insights into the usability of the app, particularly highlighting its potential as a support mechanism during times of emotional distress. One participant explained: “When I am at the point of you know getting down emotionally, I’d want to use it right before getting to that point.” Another student shared how there can be distressful moments in life and how the TE-SPI could help her get distracted from unhelpful and negative thoughts:

Sometimes there are moments where I need to de-stress or ... I need to distract myself from one specific thought or reoccurring thoughts, like flashbacks from the past and stuff ... But it [the BRITE app] would be a good distraction for me ... to distract my negative thoughts to something maybe useful.

Another theme to emerge involved perceptions that the BRITE app can serve to augment and/or replace traditional treatment contacts and/or other forms of social support. For example, one participant noted:

*I think we believe that going to therapy is more than enough, but maybe this could be an additional tool to therapy ... You know, therapist aren’t available 24 hours a day ... so this would be a helpful tool in those times.*

Another student stated:

*If I don’t have like a social support system that I can rely on at that moment, and if I feel like I need to somehow reframe my thoughts or ... just ease the mind a bit from like the negative stuff that’s going on in my mind, I will use it personally.*

However, other students felt differently. Some stated that they would not want to engage with the tool if they were having low mood. One student stated:

*When I want to release my stress ... my emotions ... I want to tell someone how I feel ... no one wants to just look at a [digital tool] like when they’re sad.*

Another participant explained that they would not choose to use the app during joyful moments, as it would be a reminder of times when they weren’t happy, stating: “I guess I wouldn’t want to use it when I’m happy because it would remind me of all the times, I was not happy.”

Overall, students highlighted various reasons as to when they would feel more inclined to use a digital mental health tool, with some students reporting that they would use it during moments of distress, while some reporting that they would avoid the tool when their mood and levels of distress improved. The consensus among participants suggests that they would use the tool sporadically, primarily as a tool for managing negative emotions. To exemplify this topic, a participant stated the following:

*I could see myself using it once a week or so. I mean like I said, maybe I should be doing more and more daily mental health check-ins, but generally, I do like self-help when I’m like feeling bad. So, when I’m feeling good, I don’t know if I would need it.*

The student feedback suggests that the TE-SPI components, including the BRITE app, may be a valuable tool when traditional social support is unavailable, as it helps reframe negative and unhelpful thoughts. However, the tool’s utility appears to be situational, with students reporting that they would be less inclined to use it when they are in a positive state.

#### Interactive Components

Participants expressed a willingness to use the app to explore and engage with suggested coping activities such as practicing mindfulness, including an affective savoring the moment activity and breathing exercises, as well as tracking their mood and distress. A participant noted the following: “Yeah, I think this could be good, like a daily check-in and like resources here but I think I would be more inclined to use it if I could customize it more.” Another participant highlighted the significance of the activities, stating:

*I think breathing exercises would help a lot. And then ... maybe the meditation part. I think the activities would probably be the main reason why I use it [the BRITE app] and the mood tracker. I think tracking your mood is really important.*

Overall, most students reported that they would use the tool primarily to practice coping skills to reduce distress, with a special interest in activities focused on meditation or mindfulness and improving sleep quality.

### Attitude Toward TE-SPI and Recommendations

During the qualitative interviews, participants were asked what would motivate them to use the app and if they would incorporate the tool into their daily lives. Many students answered that incentives and interactive games would further motivate them to engage with the digital tool. Furthermore, although the app currently includes badges that are administered based on participation, a few students suggested that incentives would be a great way to motivate and reinforce users to continuously use the tool since it could promote a sense of accomplishment and reward. For instance, a participant suggested that users could accumulate rewards over time based on tool use: “I think you would need something to get them [users] to use it more often ... gift cards or little things here and there ... maybe if they use the [BRITE app] this many times and it adds up over time, this is the amount they get.”

Similarly, another participant shared:

*I know people tend to get bored of apps. Unless it’s like a social media app ... Maybe you could add games ... Maybe you could have a leaderboard or something.*

Some participants suggested the value of adding a live chat and journaling feature that could increase usability. For example, a participant stated:

*I like to journal a lot. I do journal on my iPad, and I do write on my phone in the notes because I have everything connected, so can I just go back and forth. But that’s what would motivate me to use it.*

Others expressed that a live chat feature for crisis support would be great for users, especially if they are unable to get a hold of their mental health provider, and the live chat feature could help them to connect to a crisis hotline volunteer who could help users in times of distress. An example of this feedback is: “... if you guys do end up adding the live chat, I think it will be a good app ... either just to talk to someone ... or ask for advice ...” Overall, the participants indicated that the integration of incentives and interactive features (eg, games, journaling, and live chat) would enhance their motivation to use the app.

## Discussion

### Principal Findings

This study contributes to the growing literature on digital mental health interventions by conducting a mixed methods evaluation of college students’ perceptions of a novel, multicomponent, TE-SPI, intended for diverse college students. Unlike many mobile mental health apps that offer only standalone self-monitoring or psychoeducation, the BRITE app prototype was designed to integrate safety planning, personalized coping tools, and mood tracking, features rarely combined in a single app or digital tool, particularly for underserved student populations. A key distinction of the TE-SPI model is the integration of both the BRITE app with caring contact text messages, adding a “human touch” that may enhance perceived engagement and relevance. Using the TAM framework with the overarching aim of examining perceived usability and utility of the TE-SPI components and a qualitative rapid coding analysis methodology, our study identified salient themes related to student perceptions and preferences for optimal design features for community college students. Our study intentionally selected a diverse sample of community college students with varying levels of anxiety and depression to focus on those at elevated risk for developing future suicidal thoughts and behaviors, allowing us to examine how students at elevated risk perceived these resources during the prototype demonstration. Although participants reported possible elevated symptoms of depression and anxiety, this study was not designed or powered to examine whether symptom severity influenced perceptions of the intervention. Therefore, future research should explore whether differences in depression and anxiety symptoms are associated with actual use, usability, and perceived usefulness of digital mental health tools.

Our findings align with prior work, suggesting that students value apps that are accessible, low-burden, and offer features such as mood tracking, skill reminders, and personalization [55]. Participants indicated a perceived willingness to use a tool like the BRITE app, particularly during times of stress, for activities such as mindfulness or meditation and mood tracking. However, they also noted that their anticipated use of this particular app would likely vary depending on their individual circumstances, such as being too busy with other responsibilities. This underscores the importance of designing features that align with users’ daily needs and preferences, as well as considering strategies that may support sustained use in future real-world testing. The feedback from students regarding the app’s look and feel emphasized the importance of user-friendliness and a desire for customization features, underscoring the observation that different students are likely to use the app differently and desire different features, including alternate approaches to presenting the mood monitoring descriptors, personalization features (eg, calendar, journaling, and dark mode options), interactive games to motivate app use, and live chat functionality that could link students to other resources such as the crisis hotline. Satisfaction ratings with the BRITE app components were high, as previously found in a sample of hospitalized suicidal adolescents [25]. Still, ongoing evaluation of user engagement metrics and satisfaction surveys will be important for future optimizations to ensure that the app remains relevant and effective in supporting the mental health of college students. Because participants in this study did not use the app over time, these ratings should be interpreted as reflecting initial impressions of the prototype rather than sustained engagement or real-world use.

Participants emphasized the importance of culturally responsive content and ease of integration into daily routines. Notably, the caring contacts were especially well received, echoing prior research suggesting that students benefit from brief, nonintrusive messages that convey connection and care [56]. Importantly, participants responded to example or proposed caring contact messages rather than receiving caring contacts as part of the intervention. Underscoring the value of a “human touch,” participants felt it would be important to ensure that these messages felt authentic and human-driven, rather than automated or impersonal, to foster a sense of connection and trust among users. In addition, participants provided examples of caring contact messages that could be implemented in future caring contact interventions with this population. Participants’ feedback regarding language translation options reflects the potential need to accommodate diverse linguistic backgrounds and highlights the importance of accessibility and inclusivity. Additionally, incorporating culturally and developmentally relevant content and resources could further enhance user engagement and relevance for diverse college students. It could also promote inclusivity within the app for ensuring that users feel both represented and supported in this digital space.

### Limitations and Future Directions

While this study offers formative insights into the initial perceptions of 2 of the TE-SPI components (BRITE app and caring contacts) among ethnoracially diverse college students, several limitations should be noted. First, although the sample was diverse and sufficient to provide contextually rich preliminary feedback, it was relatively small and drawn from one segment of the transfer student or community college student population, which may limit generalizability to broader student populations. Second, participants did not download the BRITE app and did not actually receive the caring contact text messages over time as part of the study; rather, they interacted with a guided demonstration and provided feedback based on brief exposure to a prototype. Accordingly, findings reflect initial perceptions of the proposed intervention components rather than real-world feasibility, engagement, acceptability, or clinical benefit. This is particularly important, given prior findings that show app use tends to decline over time, even among students who initially express interest [56]. Additionally, the quantitative assessment in this study focused primarily on the BRITE app, with feedback on caring contacts derived largely from the qualitative interview data and only a single survey item. Therefore, conclusions regarding caring contacts are especially preliminary, and future research should evaluate this component using more robust quantitative approaches. Additional research is also needed to clarify student reactions to the clinician-guided coping or safety planning session during which the app was populated, and perceptions of this intervention component designed to offer a “human touch.” Third, the study did not assess longitudinal app use or how mental health symptoms impacted perceptions of the intervention. Without data on repeated use, in-the-moment engagement, or clinical impact, conclusions about the intervention’s effectiveness and acceptability in the real-world remain limited. This study also was not designed to estimate intervention effects or to provide effect size estimates for powering a future effectiveness trial. Instead, its primary contribution is formative and allowed for the identification of user-perceived barriers, facilitators, and priorities for refinement that can guide the next implementation phase. Incorporating ecological momentary assessment, via daily mood ratings in the app, and use analytics will be key to understanding how students engage with different features over time [57] and can help evaluate whether sustained use reduces suicide risk or improves mental health functioning among college students. Finally, while participants offered actionable recommendations to enhance personalization (eg, language translation, gamification, and live chat) during the qualitative interviews, these features were not part of the current prototype. Iterative development and testing of these enhancements may increase user engagement and cultural relevance, particularly for students from historically underserved backgrounds. As prior research has shown, users’ trust and sustained engagement are influenced not only by app content and functionality but also by transparency about data use, perceived credibility, and institutional endorsement [58]. Future work should focus on establishing trust with participants on how data collection is stored to reinforce transparency and ethical standards, ultimately enhancing intervention relevance, acceptability, and long-term impact on student mental health. Importantly, the qualitative findings highlight specific design and implementation issues, such as preference for customization features on the BRITE app or authentic caring contact text messages, that can be incorporated into the next iteration of the TE-SPI and evaluated prospectively in a more rigorous trial. More broadly, future studies should move beyond prototype feedback to test the intervention as delivered, including whether students download the app, engage with it over time, receive caring contact messages, and find the full provider-supported intervention acceptable and useful in real-world settings.

### Conclusions

This study provides preliminary feedback on a TE-SPI featuring the BRITE app—a mobile app prototype supporting coping skills use and proposed caring contact text messages among a sample of college students, who often have high unmet mental health needs and low access to treatments. In line with recent recommendations [15], TE-SPI was designed to be low-burden, contextually relevant, and responsive to the specific challenges faced by structurally marginalized students, including limited access to culturally competent care and high academic stress. The high satisfaction and perceived usability reported in response to the prototype demonstration suggest that this approach may warrant further refinement and testing as a digital intervention that could complement traditional mental health services by providing immediate, accessible, and scalable support. Given the persistent challenges in accessing mental health care on college campuses, integrating digital approaches such as the study TE-SPI into campus service infrastructures—such as counseling centers, health centers, peer support programs, and crisis response services—may be worth exploring once the intervention has been tested under real-world conditions. Moreover, the TE-SPI was designed with considerations for cultural responsiveness, accessibility, and implementation in low-resource settings [59]. Future research is needed to test the TE-SPI in a larger, more diverse student population and to evaluate actual use, acceptability, feasibility, and its impact on risk for suicidal and self-harm thoughts and behaviors. Collaboration with universities, mental health professionals, and technology developers will be key in further developing the TE-SPI approach and scaling digital interventions for broad adoption. By leveraging digital solutions like TE-SPI, mental health care providers can extend their reach beyond clinical settings, offering timely and evidence-based support to students when and where they need it most. If effectively integrated into real-world mental health services, such approaches have the potential to become a critical component in improving mental well-being and reducing suicide risk in vulnerable college populations.

## Supplementary material

10.2196/79537 Multimedia Appendix 1 Technology-enhanced suicide prevention intervention semistructured interview questions.

10.2196/79537 Multimedia Appendix 2 Technology-enhanced suicide prevention intervention interview rapid analysis coding template.

10.2196/79537 Multimedia Appendix 3 Distress mood rating and activity recommendation on the BRITE app.

10.2196/79537 Multimedia Appendix 4 Additional caring contacts text messages suggested by participants.

## Abbreviations

ELAC: East Los Angeles Community College
GAD-2: Generalized Anxiety Disorder 2-Item
PHQ-2: Patient Health Questionnaire 2-Item
SITB: self-injurious thought and behavior
STAND: Screening and Treatment for Anxiety & Depression
TAM: technology acceptance model
TE-SPI: technology-enhanced suicide prevention intervention
UCLA: University of California, Los Angeles
