# Supplementary material for: Preliminary Support for a Technology-Enhanced Suicide Prevention Intervention for College Students: Mixed Methods Preimplementation Study
Source: JMIR Hum Factors. 2026 Sep 9;13:e79537. doi: 10.2196/79537 (PMC13557321; doi:10.2196/79537)
Supplement: Supplementary file 1 [file humanfactors-v13-e79537-s001.docx]

Qualitative Questions

Introduction/Icebreaker: Hi, my name is ______. I am working with Drs. Meza and Asarnow to provide better mental health treatment for college students struggling with distressful emotions. We’d like to thank you for agreeing to share your thoughts and ideas about this. We want to remind you that your participation is voluntary, and you can end the interview any time you wish. You may also choose not to answer a question that you do not want to answer. The goal of today’s meeting, which we expect to last about 60 to 90 minutes, is to explore your opinions and feedback on a new approach to helping individuals to cope effectively and safely with high levels of stress and distressing emotions.

First, we will be asking you to complete a brief survey so we can learn about you. Once you have completed the survey, we will start the interview and will show you the BRITE+ app.

The BRITE+ app is intended to be used with college students who may be experiencing high levels of psychological distress or suicidal ideation. The BRITE+ app is intended to be introduced to a client/college student by their mental health provider. We value your opinion and want to use your ideas in the development of this new approach to improving well-being, health, and mental health.  We will be using an audio recorder and will let you know before we start recording. As a reminder, this is an anonymous interview, meaning no one will know you spoke to us or what you said.

Once we finish the interview, you will be instructed to complete a brief exit interview.

Do you have any questions? Alright, let’s get started.

*At this point, the participant will be provided their participant ID and will be instructed to complete the initial survey on the iPad.*

*Once the participant completes the survey, they should be made aware that the interview will begin and the audio recorder will be turned on.*

*The interview will reflect a semi-structured format where participants will be prompted to provide feedback on the topics listed below. This format will allow for flexibility as new topics may arise. Based on participant feedback, the interviewer should follow up as appropriate with additional questions to clarify or elicit any additional information. Questions may also be skipped to manage participant burden and ensure that the interview is not overly long*. *Select probes/questions and follow-up to clarify and build on participant comments, and areas of focus based on progress in qualitative data collection.*

[Interviewer will show participant the BRITE+ app on an IPAD or will show depictions of the app features]

1. What are your initial thoughts about the BRITE+ app that I just showed you?
2. Would you actually want to use the BRITE+ app?
3. What are the situations where you think you would choose to use the BRITE+ app? Are

there any situations where you wouldn’t want to use it?

4. Do you think the BRITE+ app would be helpful in moments when you may feel distress?
5. Based on the mock-up of the BRITE+ app, do you have any suggestions for making the

app easier to use? We are asking about ways in which we could improve the flow or

function of the app, e.g., how to enter or navigate the app to find desired information.

6. Based on the mock-up of the BRITE+ app, do you have any suggestions for improving

the look & feel of the app? We are asking about colors, font, images, etc. that would

make you want to use BRITE+.

7. Based on the mock-up of the BRITE+ app is there any content that you would add or

remove? *Prompt: If there was a section of the app listing these different skills, what*

*would your initial reaction be? (show pictures of the different skills)*

8. What would motivate you to use the BRITE+ app as a regular part of your daily routine?

How long do you think you would you use the BRITE+ for? (days, weeks, months)

9. What, if anything, do you think would get in the way of using the BRITE+ app?
10. Would you be worried about confidentiality or privacy using the BRITE+ app? Why or

why not?

11. Overall, do you feel the content and language is relevant to your cultural group? (Note:

cultural group includes people that share similar values to you, those who may share

similar traditions or a group you identify strongly with).  If no, what would you change?

If yes, what were the aspects you liked?

12. Do you feel that the language and content is appropriate for college-age students? If yes,

what in particular? If no, what you would change?

13. We know that sometimes individuals don’t use apps or use them less than they thought

they would, even if they like the app or found it helpful. Do you see this as a problem?

Do you think there is anything that could work for helping to encourage app use?

14. Are there any other comments or questions you would like to share about BRITE +?
