# Supplementary material for: Preliminary Support for a Technology-Enhanced Suicide Prevention Intervention for College Students: Mixed Methods Preimplementation Study
Source: JMIR Hum Factors. 2026 Sep 9;13:e79537. doi: 10.2196/79537 (PMC13557321; doi:10.2196/79537)
Supplement: Supplementary file 2 [file humanfactors-v13-e79537-s002.docx]

**TE-SPI Interview Rapid Analysis Coding Template**

Participant ID#: XX

STEP 1:  Create a neutral domain name that corresponds with each interview question.

| Construct | Question | Domain | Quote(s) |
| --- | --- | --- | --- |
| First Impression | What are your initial thoughts about the BRITE+ app that I just showed you? |  |  |
| Usage | Would you actually want to use the BRITE+ app? |  |  |
| Situational Usage | What are the situations where you think you would choose to use the BRITE+ app? Are there any situations where you wouldn’t want to use it? |  |  |
| Distress Use | Do you think the BRITE+ app would be helpful in moments you may feel distress? |  |  |
| Functionality | Based on the mock-up of the BRITE+ app, do you have any suggestions for making the app easier to use? We are asking about ways in which we could improve the flow or function of the app, e.g., how to enter or navigate the app to find desired information. |  |  |
| Design Appeal/Functionality | Based on the mock-up of the BRITE+ app, do you have any suggestions for improving the look and feel of the app? We are asking about colors, font, images, etc. That would make you want to use the BRITE+ app. |  |  |
| Content/Functionality | Based on the mock-up of the BRITE+ app is there any content that you would add or remove? |  |  |
| Usage | What would motivate you to use the BRITE+ app as a regular part of your daily routine? How long do you think you would use the app for? (days, weeks, months) |  |  |
| Usage (Interface) | What, if anything, do you think would get in the way of using the BRITE+ app? |  |  |
| Confidentiality | Would you be worried about confidentiality or privacy using the BRITE+ app? Why or why not? |  |  |
| Cultural & Age Relevancy/ Connectiveness | Overall, do you feel the content and language is relevant to your cultural group? (Note: cultural group include people that share similar values to you, those who may share similar traditions or a group you identify strongly with). If not, what would you change? If yes, what were the aspects you liked? |  |  |
| Cultural & Age Relevancy/ Connectiveness | Do you feel that the language and content is appropriate for college-age students? If yes, what in particular? If no, what you would change? |  |  |
| Usage (Interface) | We know that sometimes individuals don’t use apps or use them less than they thought they would, even if they like the app or found it helpful. Do you see this as a problem? Do you think there is anything that could work for helping to encourage app use? |  |  |
| Caring Message Approach | What do you think of this approach? |  |  |
| Message preferences | What would you want to see in these messages? |  |  |
|  | What should definitely not be in these messages? |  |  |
| Message Examples | Can you share a message that you would like to receive from a mental health provider? |  |  |

STEP 2: Draft a summary template for use by the team.

TRANSCRIPT SUMMARY

PREPARED BY:

SITE:

RESPONDENT ROLE:

OVERALL SUGGESTIONS

EFFECTIVENESS OF BRITE+

RESPONSE TO BRITE+ FORMAT

OTHER OBSERVATIONs
