# Supplementary material for: Preliminary Support for a Technology-Enhanced Suicide Prevention Intervention for College Students: Mixed Methods Preimplementation Study
Source: JMIR Hum Factors. 2026 Sep 9;13:e79537. doi: 10.2196/79537 (PMC13557321; doi:10.2196/79537)
Supplement: Supplementary file 3 [file humanfactors-v13-e79537-s003.docx]

**Distress Mood Rating and Activity Recommendation on the BRITE App**

References

Kennard BD, Goldstein T, Foxwell AA, McMakin DL, Wolfe K, Biernesser C, et al. As Safe as	Possible (ASAP): A brief app-supported inpatient intervention to prevent postdischarge	suicidal behavior in hospitalized, suicidal adolescents. *American Journal of Psychiatry*.	2018 Sep 1;175(9):864–72. PMID: 30021457
