# Supplementary material for: Preliminary Support for a Technology-Enhanced Suicide Prevention Intervention for College Students: Mixed Methods Preimplementation Study
Source: JMIR Hum Factors. 2026 Sep 9;13:e79537. doi: 10.2196/79537 (PMC13557321; doi:10.2196/79537)
Supplement: Supplementary file 4 [file humanfactors-v13-e79537-s004.docx]

**Table S1**. Additional Caring Contacts Text Messages Suggested by Participants

| **Suggestions** | **Participant** |
| --- | --- |
| “You’re strong. You can do this!” | Female, 20, Transfer Student |
| “Remember even if you are going through tough times, it gets better.” | Female, 21, Transfer Student |
| “Hey, I hope you are having a great day today.” | Female, 31, Community College Student |
| “Keep up the good work!” | Male, 30, Community College Student |
| “Hey, just thinking of you.” | Female, 22, Community College Student |
| “Do your best!” | Male, 20, Transfer Student |
| “Today’s a new day, and you’re going to do well!” | Female, 20, Transfer Student |
